# The promise and pitfalls of monitoring harmful algal blooms (HABs) with remote sensing in pond-sized waterbodies

**DOI:** 10.1007/s11356-026-37902-w

**Published:** 2026-06-05

**Authors:** Nayoung Joyce Hur, Keith Steven Jennings, Diane Marie McKnight

**Affiliations:** 1 Department of Civil, Environmental, and Architectural Engineering, University of Colorado Boulder, Boulder, CO USA; 2 Lynker Corporation, Boulder, CO USA; 3 University of Vermont Water Resources Institute, Burlington, VT USA

**Keywords:** Harmful algal blooms, HABs, Remote sensing, Satellite validation, Management, Chlorophylla, Limnology

## Abstract

Harmful algal blooms (HABs) pose water quality risks, including the depletion of dissolved oxygen and human health impacts. Remote sensing is a proven tool for monitoring HABs, yet knowledge is limited about its effectiveness in pond-sized waterbodies, whose size and shape may preclude multi-spectral platforms with large spatial resolutions and increase the probability of mixed pixels. This comparative limnology case study evaluates whether optical remote sensing is a viable tool to monitor HABs in pond-sized waterbodies. We use Sentinel-2 imagery with previously studied chlorophyll-a and cyanobacteria detection algorithms and performed targeted in situ sampling in four small waterbodies in Boulder, CO, USA, from June to August 2021 to validate the algorithms and better understand underlying biogeochemical processes. The chlorophyll-a algorithm indicated persistent algal growth occurred in all waterbodies, yet only Sombrero Marsh chlorophyll-a expressed a statistically significant relationship with the remote sensing output (*p* < 0.0005, *r*^*2*^ = 0.80). Meanwhile, the cyanobacteria algorithm resulted in false negatives, only showing potential cyanobacteria at Sombrero Marsh despite in situ samples from all waterbodies indicating cyanobacteria were present. Samples from Sombrero Marsh had the highest chlorophyll-a (average = 132.5 µg/L) and percent cyanobacteria (average = 43.5%). These findings suggest that there is uncertainty in relying on remote sensing for monitoring HABs in small waterbodies unless a high concentration of algae is present on the water surface. However, in a resource- and time-limited system, remote sensing can be a useful tool as an initial assessment for monitoring algal blooms.

## Introduction

Small inland waterbodies, including ponds, can be valuable community resources. In a recent analysis, a functional definition of ponds was developed to distinguish ponds from small lakes and wetlands based on their area (< 5 ha), depth (< 5 m), and less than 30% coverage by emergent vegetation (Richardson et al. 2022). Ponds can be productive, biogeochemical hotspots on a landscape (Rabaey et al. 2024) and can provide habitat for wildlife and numerous ecosystem services like drinking water supply, recreation outlets, and hazard buffers (e.g., flood and drought mitigation) (Grizzetti & Poikane 2024). Algal growth is a key function in pond ecosystems, as these microorganisms produce oxygen through photosynthesis and serve as food for zooplankton, and thus fish and wildlife (Chapman 2013; Stevenson 2014). The small surface area and shallow depths of ponds can promote internal nutrient cycling and enhance algal growth (Richardson et al. 2022). With this nutrient loading and rising water temperatures, algal blooms can develop (Fernandez-Figueroa et al. 2024; Gobler 2020; Paerl & Paul 2012). In some cases, these blooms may be harmful algal blooms (HABs) that pose risks to ecosystems, such as depleted oxygen levels from algal biomass decomposition, high respiration demands, and/or the presence of toxins harmful to aquatic life, animals, and humans.

HABs lead to myriad negative outcomes, including fish kills (Landsberg et al. 2020; Rodger et al. 1994), adverse health impacts to wildlife and pets such as illness and death from ingesting elevated levels of cyanotoxins (Backer et al. 2013; Hilborn & Beasley 2015), and human health issues such as respiratory problems, skin irritation, or impacts to the nervous system (Sonak et al. 2018). Most human and wildlife health risks in freshwater systems arise from the overabundance of cyanobacteria, which are harmful if they release cyanotoxins in the affected waterbody (Backer et al. 2013). This concern is unique to cyanobacterial HABs (cHABs) as several, but not all, cyanobacteria taxa have secondary metabolites that can result in release of toxins during cell lysis (Bramburger et al. 2023; Merel et al. 2013).

Environmental stressors associated with climate change, such as warming-induced eutrophication (Gobler 2020; Moore et al. 2008), and anthropogenic influences, such as nutrient enrichment, work in tandem to promote bloom development and furthermore complicate bloom management (Carey et al. 2012; Griffith & Gobler 2020; Nwankwegu et al. 2019). In essence, algal growth requires light, water, nutrients, and carbon dioxide, so warmer waters in parallel with higher solar irradiance, during spring to fall months, yield more opportune conditions for algal growth. These warmer water temperatures may be associated with longer residence times resulting in stagnant and stratified waters (Clark et al. 2017; Moore et al. 2008; Paerl et al. 2018). In freshwater, climate change–induced temperature increases have shifted algal assemblages, generally favoring cyanobacterial growth at higher water temperatures (Bonilla et al. 2023; Carey et al. 2012). Additionally, because cyanobacteria can regulate their buoyancy, cyanobacteria growth is mechanically favorable in lower viscosity and increased stratification waters (EPA, 2022). These effects are also nuanced depending on the cyanobacterial genera (e.g., *Anabaena* or *Microcystis*), as some genera respond differently to nutrient, salinity, and carbon dioxide (CO_2_) concentrations. Nonetheless, warming temperatures and nutrient loading tend to produce more intense, frequent, and longer-lasting algal blooms.

To mitigate negative impacts on water quality and to protect human and ecosystem health, it is critical to monitor algal bloom intensity and timing in inland waterbodies. One approach is to use remote sensing platforms, which provide near-real-time surface reflection data that can be used to evaluate spatiotemporal patterns of algal blooms. The advantage here is that the data are continuous, frequent, and freely available. While studies have demonstrated that remote sensing analyses can determine the frequency and timing of algal growth and HABs in natural waters using various algorithms (Cao & Han 2021; Ho & Michalak 2017; Karki et al. 2021; Khan et al. 2021; Oyama et al. 2015), a recent review has noted that there are limits to the applicability of remote sensing data (Wu et al. 2025) associated with limited spatial resolution which determines the size of waterbodies that can be evaluated. These challenges are acute for small waterbodies, such as ponds, because the large pixels of some remote sensing platforms overlap with the pond’s edge, producing mixed pixels that include land and water surfaces (Liu et al. 2022). Pixels near the edge may include emergent macrophytes leading to false detections of algal blooms.

A complementary approach to remote sensing is in situ sampling, which can provide valuable information not only about the presence of specific types of algae including cyanobacteria, but also about water quality parameters including pH, temperature, dissolved oxygen, conductance, and nutrient concentrations. These in situ measurements can reveal the environmental conditions that drive bloom formation and persistence (Bramburger et al. 2023). Subsequent laboratory analyses, such as measuring chlorophyll-a concentrations and determining the types of organisms present in a sample via microscopy, allow for quantification of algal prevalence (Dörnhöfer & Oppelt 2016). Nonetheless, in situ sampling can be resource intensive and may involve delays in sample processing, limiting a thorough, timely analysis of HABs and cHABs as these blooms are easily disturbed and sensitive to many meteorological factors (Kutser et al. 2008; Zhao et al. 2020). Thus, complementing remote sensing products with in situ sampling provides a more robust evaluation of algal growth, potential cHABs, and algal composition.

In Colorado, USA, news reports and state agency data from the past several years indicate the emergence of HABs as a public health and water quality concern (Brown 2020; Colorado Department of Public Health 2020). Colorado depends heavily on inland waterbodies for drinking water and recreation. HAB reports have spurred in situ monitoring campaigns conducted by Colorado Parks and Wildlife (CPW) staff, but the sample sites are a selective group of about 70 waterbodies out of the thousands spread across the state. In response, other groups including the City of Boulder Open Space and Mountain Parks (OSMP) have leveraged remote sensing to monitor a larger proportion of their managed waterbodies, which includes numerous pond-sized waterbodies. Their first study indicated that algal growth has increased in the past decade relative to the 1990 s and 2000 s and that HABs peak in the summertime when water temperatures are higher, solar radiation is greater, and spring runoff has ceased (Jennings et al. 2020). However, a shortcoming of this study was the lack of validation data to evaluate the remote sensing output.

Few previous studies have evaluated the relationship between in situ data and remote sensing retrievals in small- to medium-sized inland waterbodies (Zabaleta et al. 2021; Kislik et al. 2022; Liu et al. 2022; Lopez Barreto et al. 2024) such as those managed by Boulder OSMP. In particular, Lopez Barreto et al. (2024) concluded that remote sensing may serve as an effective early indicator for possible exposure advisories for cHABs, making it a trigger for additional in situ sampling. The common theme that emerges is the necessity of validating remote sensing outcomes with water sampling data when using the former to augment information from the latter. Here, we examine the promise and pitfalls of using remote sensing to evaluate algal blooms in pond-sized waterbodies based upon comparison to in situ data. We hypothesized that remote sensing would provide a useful indication of HAB and cHAB formations despite the remote sensing limitations. Our primary objective was to employ novel lab-based and computational methods to assess the accuracy of remote sensing estimates of algal growth and HAB presence in these small waterbodies that can be important environmental and recreational resources for the local community.

## Materials and methods

To evaluate algal blooms and the presence of cyanobacteria in the selected ponds, we developed a multi-step remote sensing and in situ sampling protocol (Table 1). We provide a brief overview here, while further details follow in the subsequent sections: (1) we collected biweekly water samples in four inland waterbodies managed by Boulder OSMP during the summer months of 2021; (2) we ran the remote sensing analysis for each waterbody in our domain; (3) we analyzed the water samples for the parameters of interest, e.g., chlorophyll-a and nutrient concentrations, and analyzed the phytoplankton samples through the FlowCam, which is a digital imaging flow cytometer designed to rapidly process and identify algal species in a water sample; and (4) we related the measured parameters to the remote sensing output. To this last point, as the abundance of algae increases, so does the concentration of chlorophyll-a in a water sample and previous research has shown that the remotely sensed NIR:Red ratio expresses a positive relationship with chlorophyll-a concentrations (Watanabe et al. 2015; Yu et al. 2014).

**Table 1:** Variables used in this analysis as derived from in situ, lab, and remote sensing data

Variable	Unit	Instrument	Source	Description and Justification
Temperature	°C	Probe	In situ	Water temperature; to assess conditions conducive to cyanobacterial growth
pH	-	Probe	In situ	Acidic/basic condition; to assess conditions conducive to algal growth
Specific conductance	µS/cm	Probe	In situ	Measure of collective dissolved ions in the water (at a standard 25ºC); to assess conditions conducive to algal growth
Dissolved oxygen	mg/L%	Probe	In situ	Measure of oxygen saturation for a given temperature; to assess influence of algal productivity
Chlorophyll-a and phaeophytin	mg/L	FluoroMax-3	Lab	Concentration of chlorophyll and phaeophytin; to assess algal biomass and condition
Algae type	-	FlowCam	Lab	Type of algae determined through visual identification of FlowCam images
Cyanobacteria abundance	-	FlowCam	Lab	The absolute and relative number of cyanobacteria in a 5 mL FlowCam sample
Nutrients (NO_3_^2−^, NO_2_^−^, NH_4_^+^, SRP)	µg L^−1^	Ion Chromatography (IC) and Seal AA3	Lab	IC measured the nitrate and nitrite; Seal AA3 measured the ammonia and soluble reactive phosphorous (SRP); to assess nutrient availability to support algal growth
NIR:Red ratio	-	Sentinel-2	Remote sensing	Band ratio of near infrared (NIR) to red that provides a continuous range of values that are indicative of algal concentration
FAI-NDWI	-	Sentinel-2	Remote sensing	A two-step algorithm that combines the floating algae index (FAI) and the normalized difference water index (NDWI) to evaluate cyanobacteria as a binary presence/absence

### Study area

**Fig. 1:**
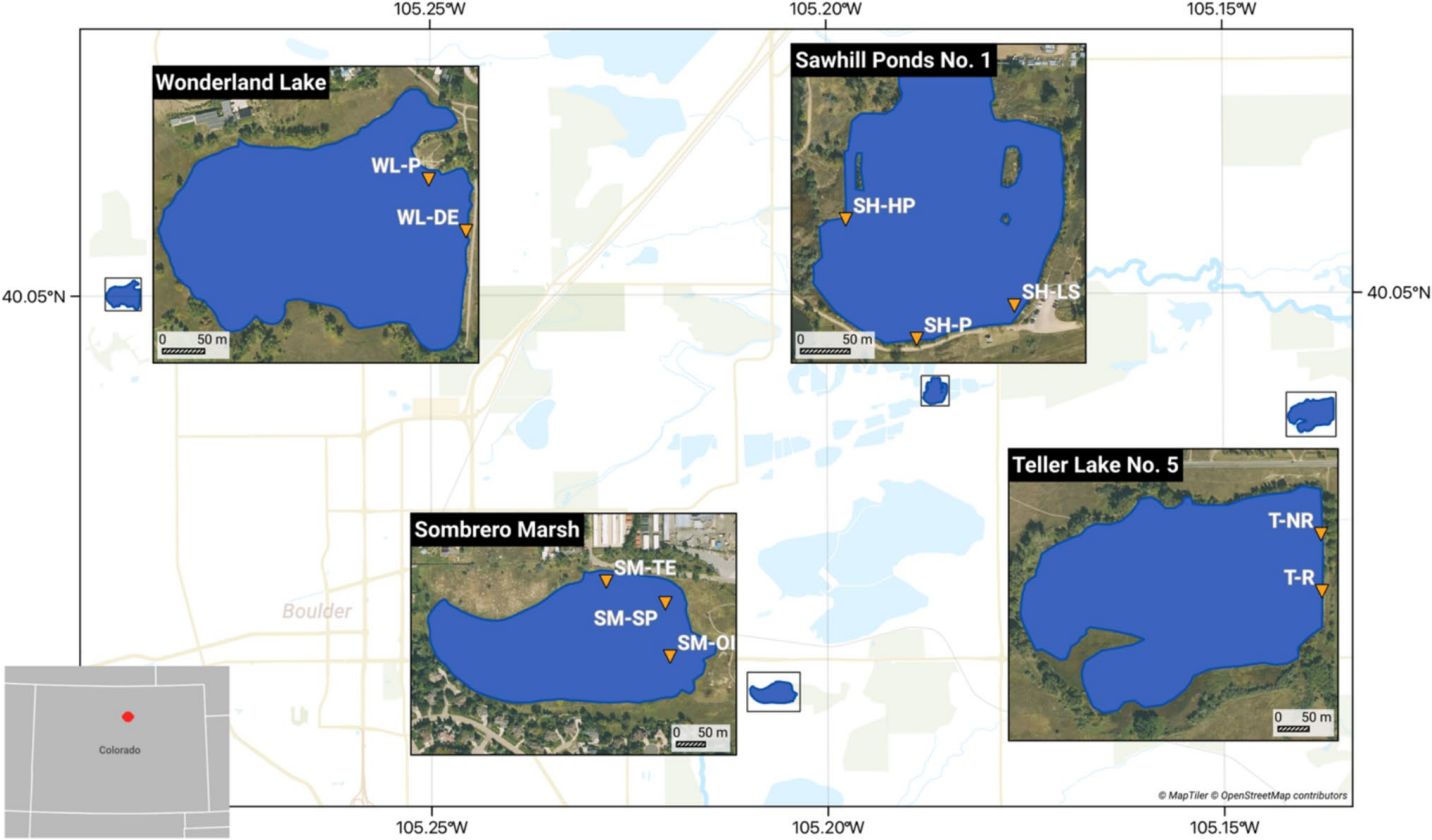
Map of the study domain in Boulder County, Colorado. The four sample sites are highlighted for each waterbody. The sample locations are noted in the orange pins; each sample location is tagged with an abbreviated naming convention for the waterbody. Each name is structured as follows: the abbreviated name of the waterbody, followed by a hyphen, and then the starting initials of the sampling location. For example, SH-LS refers to Sawhill Loading Shore. Note: the waterbody boundaries are sourced from the OSMP database and are used for cartographic reference only; they may not accurately represent true water extents

We evaluated summertime algal blooms in four pond-sized waterbodies in Boulder County, Colorado under Boulder OSMP management (Fig. 1). We targeted these ponds because a previous remote sensing analysis indicated they each experienced algal growth during the summer months, with some potentially experiencing a cyanobacterial bloom (Jennings et al. 2020). All selected waterbodies have the physical (i.e., surface area and depth) and biological characteristics (i.e., emergent vegetation covering less than 30% of the area) close to the range identified by Richarson et al. (2022) to qualify as ponds (OSMP, personal communication).

Sawhill Ponds No. 1 is the first of the 18 ponds in a wetland habitat. Historically, this area was a collection of sand and gravel pits, as gravel was mined until operations ceased in the early 1970s. The pond has a surface area of 4.8 ha with a max depth of 2.1 m and is primarily groundwater fed and sometimes fed by an inlet ditch. There is no observable surface outflow for this pond. Teller Lake No. 5 has a surface area of 5.3 ha with a max depth of 1.8 m and sits within farmland used for livestock grazing and crop production and serves as a reservoir for the surrounding farms and ranches. There is a dam alongside the far east shore of the pond, and the pond is fed through inlet ditches in May. Managers pass water via ditches to irrigate fields north of the pond throughout the summer. Sombrero Marsh is one of the only closed, natural basins of Boulder OSMP managed waterbodies and has a surface area of 4.2 ha with a max depth of 0.9 m. Historically, it was used as a waste pit until it was revitalized into a wetland habitat. The pond is known for its brackish, highly alkaline waters, and serves an important role in maintaining ecological diversity (OSMP, personal communication). Wonderland Lake, while it has an area of 6.1 ha, is shallow at a maximum depth of 4.8 m. Wonderland Lake is a popular recreation destination, surrounded by trail networks and residential areas to the north, east, and south. The pond is primarily fed by groundwater and some stormwater with limited outflow during springtime melt and precipitation events. Molecular analyses of algal samples from Wonderland Lake completed in the summer months of 2021 and 2022 have indicated the presence of potentially toxin-producing cyanobacteria, notably *Microcystis* and *Synechococcus* (Collins 2023).

### Remote sensing

We analyzed high-resolution optical imagery from the Sentinel-2A and 2B satellites, which offer several advantages over other instruments, including a finer spatial resolution (10 m versus 30 m for Landsat) and shorter repeat times between satellite overpasses (2 to 5 days) (Appendix Table 4). Specifically, we accessed the Sentinel-2 Level 1-C Top of Atmosphere product using the Google Earth Engine processing platform (Gorelick et al. 2017; Sentinel-2 MSI, n.d). We applied two algorithms to the Sentinel-2 data. The first algorithm ratios the reflectance of the near infrared (NIR) and red bands. The NIR:Red ratio is close to zero for clear water and increases with algae concentrations (Tebbs et al. 2013). We also used a rule-based algorithm to evaluate cyanobacterial presence using two other remote sensing metrics, the Floating Algae Index (FAI) and the Normalized Difference Water Index (NDWI):

1 $$FAI={R}_{NIR}-\left[{R}_{Red}+\left({R}_{SWIR1}-{R}_{Red}\right)\times \frac{\left(865-655\right)}{\left(1610-655\right)}\right]$$

2 $$NDWI=\frac{({R}_{NIR} - {R}_{SWIR1}) }{({R}_{NIR} + {R}_{SWIR1})}$$

where $R$ represents the top of atmosphere reflectance (0–1) for a given band (e.g., ${R}_{NIR}$) is the reflectance value for the NIR band). Compared to the simple NIR:Red ratio, the algorithm based on FAI and NDWI is slightly more complex, employing two thresholds in a rule-based scheme to estimate the presence of a cyanobacterial bloom (Oyama et al. 2015). First, the FAI differentiates between clear water and algae using a threshold of 0.05. The FAI considers values greater than or equal to 0.05 to be algae, while those below are clear water. Next, the NDWI partitions algae (i.e., pixels with FAI ≥ 0.05) into cyanobacteria and non-cyanobacteria blooms using a threshold of 0.63. Values greater than or equal to this threshold are considered probable cyanobacterial blooms while those below are not.

For each in situ sampling location (Fig. 1), we created three types of data extraction points: edge, near-edge, and open-water for the remote sensing imagery. This approach isolates the actual (edge) or potential (near-edge) overlap of the remote sensing imagery with the waterbody edge. We include output from all three types for two reasons: (1) the mixture of land and water in a remote sensing pixel will influence algorithm output compared to open-water pixels and (2) algae are more likely to concentrate near the shore and we performed all manual sampling from the shoreline. Once we had created the extraction points, we then used Google Earth Engine to access all Sentinel-2 data between 2021-04−01 and 2021-10−01 at these locations, removing days when clouds obscured the earth’s surface from the satellite sensor. With these data, we created a time series of the NIR:Red ratio and the FAI-NDWI cyanobacteria algorithm at each extraction point.

In addition to the time series data, we created maps for each waterbody showing the number of times each pixel exceeded a NIR:Red ratio greater than 1 and met the conditions for cyanobacteria according to the FAI-NDWI algorithm. This analysis was done in a slightly different manner than the time series data extraction. We stepped through each day of Sentinel-2 data and calculated the NIR:Red ratio and ran the FAI-NDWI algorithm across every pixel for the waterbodies. We created two new maps, one corresponding to each algorithm. We added 1 to each pixel in the maps whenever the NIR:Red or FAI-NDWI thresholds were respectively exceeded in that pixel on a cloud-free day with Sentinel-2 data. The final products were the two maps, masked to the waterbodies of interest, showing the number of times each pixel within a given waterbody exceeded a NIR:Red ratio greater than 1 and met the conditions for cyanobacteria according to the FAI-NDWI algorithm.

### Field sampling

We collected water samples from June to mid-August 2021 on a biweekly basis from accessible shoreline locations in the four ponds (Fig. 1 and Appendix Table 3). We took in situ field meter measurements at the time of collection for water temperature, pH, conductance, and dissolved oxygen using a YSI Model 556 multiparameter sonde. We collected grab samples at each site using 1.0 L clear Nalgene bottles. All Nalgene bottles were rinsed five times with ultrapure deionized water before being used in the field. To ensure representative samples, we rinsed and purged the bottle with the sampling site water twice before filling the sample bottle a final time and then the bottle was stored in a cooler. Samples for algae were aliquoted and filtered within 3 h of collection. Analysis bottles for algae composition were spiked with 1.0 mL of 5.0% Lugol solution (potassium iodine) to preserve the sample, which we then refrigerated (Herve & Heinonen 1982). Nutrient samples were filtered with combusted 0.45-µm glass fiber filters. Both the filter, used for chlorophyll-a analysis, and the filtered sample were stored in the freezer.

### Lab analyses

#### Phytoplankton composition

We determined algae type in the Lugol-preserved samples from each waterbody using flow-through imaging microscopy (FlowCam). FlowCam results have been shown to be comparable to conventional microscopy in previous research (Camoying & Yñiguez 2016). We determined algal composition by aliquoting 5 mL of the sample into the FlowCam. For our FlowCam setup, we used a 10 × objective lens which translates to 100 × magnification. We set the flow rate to 0.150 mL/min yielding a 14% efficiency, indicating that 14% of the volume was imaged. As the sample goes through the FlowCam’s flow cell, the instrument recognizes and takes pictures of the specimen. This results in a mixture of images, from algae to debris in the water, with debris making < 5% of the total count.

The FlowCam outputs individual TIFF files arranged on a grid, typically on micron scales (Appendix Fig. 9). These images were sorted by date and sampling site. Visual Spreadsheet, a program created by Fluid Imaging, Inc., provides metrics on each FlowCam run. This program estimates biovolume and type of algae once trained with classification data. The classification dataset was developed first by manually sorting through samples of FlowCam images to identify cyanobacteria as well as other organisms (e.g., green algae and dinoflagellates). We counted predominantly filaments of cyanobacteria, as some toxic cyanobacteria are filamentous. We determined counts as the number of cyanobacteria filaments and other particles (i.e., all particles, algae or not) from all sites for each waterbody on the day of collection.

#### Chlorophyll-a

All sample preparation and measurement for chlorophyll-a was performed in a darkened room to minimize the degradation of photosynthetic pigments. We filtered 100 mL of the sample water from each site through combusted glass fiber filters that we then wrapped in foil and kept frozen until 2 days before sample analysis. The frozen wrapped filters were thawed for a minimum 24 h at room temperature, and then placed in a glass vial wrapped in aluminum foil with 20 mL 90% buffered acetone and set in a refrigerator for 24 h to allow pigment extraction. We measured each sample twice with the Horiba FluoroMax-3 fluorometer, the first measurement for chlorophyll-a, and the second measurement with the solution spiked with 0.1 M HCl for phaeophytin. Phaeophytin is a compound formed with the degradation of chlorophyll-a (Owens & Falkowskit 1982). This derivative can be indicative of the senescence of algal communities with light energy being converted to chemical energy, so we expect a semi-positive relationship between phaeophytin and chlorophyll-a values; while the concentrations for chlorophyll-a and phaeophytin follow similar trends, when chlorophyll-a values are high, the phaeophytin values may be lower and vice versa.

The fluorometer outputs two intensities: ${S}_{ b}$ and ${S}_{ a}$, which are before acid and after acid, respectively. We can then compute concentrations using the following equations:

3 $$\left[Chl-a\right]\left(\frac{ug}{L}\right)=Cf\left(\frac{r}{r-1}\right)\left({S}_{b-blank}-{S}_{a-blank}\right)\left({V}_{a}/{V}_{s}\right)$$

4 $$\left[Phaeophytin\right]\left(\frac{ug}{L}\right)=Cf\left(\frac{r}{r-1}\right)\left[\left(r\cdot {S}_{a, blank}\right)-{S}_{b, blank}\right]\left({V}_{a}/{V}_{s}\right)$$

where $Cf$ refers to the calibration factor (which is the slope of ${S}_{ b}$ vs. the calculated chlorophyll concentrations of the diluted standards determined by a spectrophotometer; $Cf=0.0493$), $r$ is the average ratio of ${S}_{b}/{S}_{a}$ $(r=4.80)$, ${S}_{a-blank}$ and ${S}_{b, blank}$ are to the intensities minus the blank measured from calibration, ${V}_{a}$ is the volume of acetone added to extract the pigment (${V}_{a}=0.02 L$), and ${V}_{s}$ is the volume of the sample filtered.

We applied an additional threshold-based metric to chlorophyll-a values known as the trophic state benchmark categories to estimate biological activity in the waterbody (EPA, 2022). According to the benchmark, a waterbody is considered oligotrophic with chlorophyll-a concentrations of ≤ 2 µg/L, mesotrophic with > 2 µg/L but ≤ 7 µg/L, eutrophic with > 7 µg/L but ≤ 30 µg/L, and hypereutrophic with > 30 µg/L. This initial screening offers a preliminary indication of the waterbody’s condition based on standardized trophic state thresholds.

#### Nutrients

Water samples were thawed to room temperature and then aliquoted for nutrient analysis. Nitrate-N ($N{O}_{3}^{-}$) and nitrite-N ($N{O}_{2}^{-}$) were analyzed on an ion chromatography system (Thermo Fisher Scientific, Dionex ICS 1100). Ammonia-N ($N{H}_{4}^{+}$) and total soluble reactive phosphorus (SRP) were analyzed on an air segmented flow analyzer (SEAL AutoAnalyzer 3). Total inorganic nitrogen (TIN) is the sum of $N{O}_{3}^{-} + N{O}_{2}^{-} + N{H}_{4}^{+}$. Method-detection limits for both instruments are 1 µg/L. Values that were below the detection limits are reported with low relative precision.

Nutrient data were further analyzed using empirical nitrogen to phosphorus (TIN:SRP) relationships of 16:1 and 23:1, referred to as the Redfield ratios. Applying these Redfield ratios helps to better understand N and P deficiencies in freshwater systems (Hecky et al. 1993) and their relationship to HABs presence.

### Remote sensing to in situ data comparison

To determine the effectiveness of the remote sensing algorithms in detecting algal blooms and HABs, we evaluated both algorithms against the chlorophyll-a and algae type data. For the NIR:Red ratio output, we quantified the ratio’s mathematical relationship to the lab-determined chlorophyll-a concentrations from the water quality samples. To do this, we associated each in situ sample of chlorophyll-a to the corresponding average NIR:Red ratio value for that open water sampling location within ± 15 days of the sampling date to account for noise in the sensor data as well as infrequent valid Sentinel-2 data resulting from cloud cover and variable overpass timing. We then plotted the remotely sensed NIR:Red ratio against the chlorophyll-a concentration and computed a line of best fit to determine the mathematical relationship between the two. We additionally analyzed whether cyanobacteria detected by the FlowCam corresponded to cyanobacteria indicated by the FAI-NDWI algorithm. Here, we associated in situ cyanobacteria presence/absence (when the number of FlowCam-detected cyanobacteria exceeded 5) with whether the FAI-NDWI algorithm had indicated blue-green algae presence at any time within ± 15 days. We then computed the number of true positives (yes-yes), true negatives (no-no), false positives (yes-no), and false negatives (no-yes) of the remote sensing detection algorithm compared to the in situ data.

## Results

### Remote sensing outputs

**Fig. 2:**
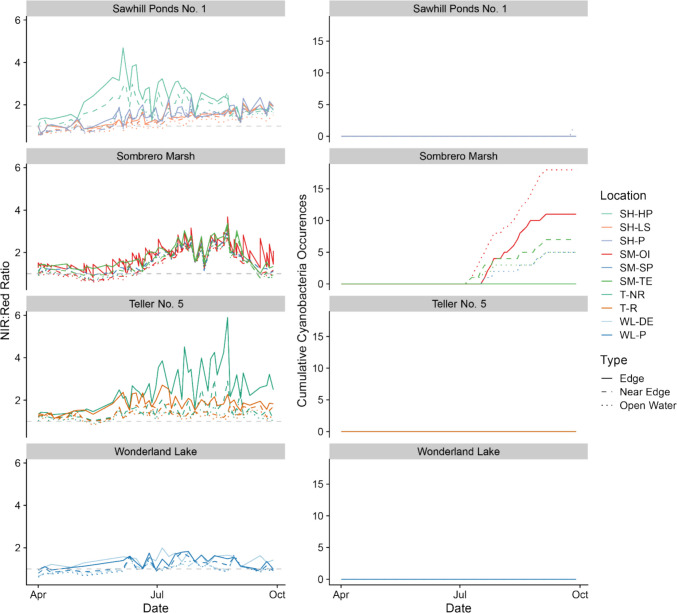
2021 remote sensing results of the NIR:Red and FAI-NDWI outputs at each waterbody. (Left) A time series of the NIR:Red ratio at each sampling location and pixel type for the four waterbodies in our analysis. The gray dashed line corresponds to a NIR:Red value of 1. Traces above this line are consistent with algae growth. (Right) The cumulative cyanobacteria occurrences based on the FAI-NDWI algorithm at each sampling location and pixel type for the four waterbodies in our analysis

According to the NIR:Red remote sensing data, all four ponds expressed evidence of algal growth during the spring and summer of 2021 (Fig. 2; Left). For each location and pixel type, the NIR:Red ratio exceeded 1 for at least several satellite overpasses. In general, the NIR:Red ratio increased from a minimum in early spring to a maximum in late summer, after which it decreased back towards the minimum. The seasonal pattern was most pronounced at Sombrero Marsh, where all locations showed a clear cycle of algal growth (increasing NIR:Red values) and senescence (decreasing NIR:Red values). The pattern was relatively muted at Wonderland Lake, indicating a lower degree of algal growth during the summer months. Open water pixels typically had the lowest NIR:Red values, indicating either greater algal growth for the edge and near edge pixels or potential effects of shoreline vegetation on algorithm output.

The findings were different for the FAI-NDWI algorithm, which indicated consistent cyanobacteria presence only at analyzed locations in Sombrero Marsh (Fig. 2; Right). Although the NIR:Red ratio increased at most sites starting in late spring, the FAI-NDWI algorithm did not show potential cyanobacterial growth until July. Also of note is the fact that open water and near edge pixels typically expressed higher cyanobacterial contents than the edge pixels, which may have been a result of the NDWI reducing edge effects by partitioning values into either vegetation (< 0.63) or cyanobacteria (≥ 0.63).

**Fig. 3:**
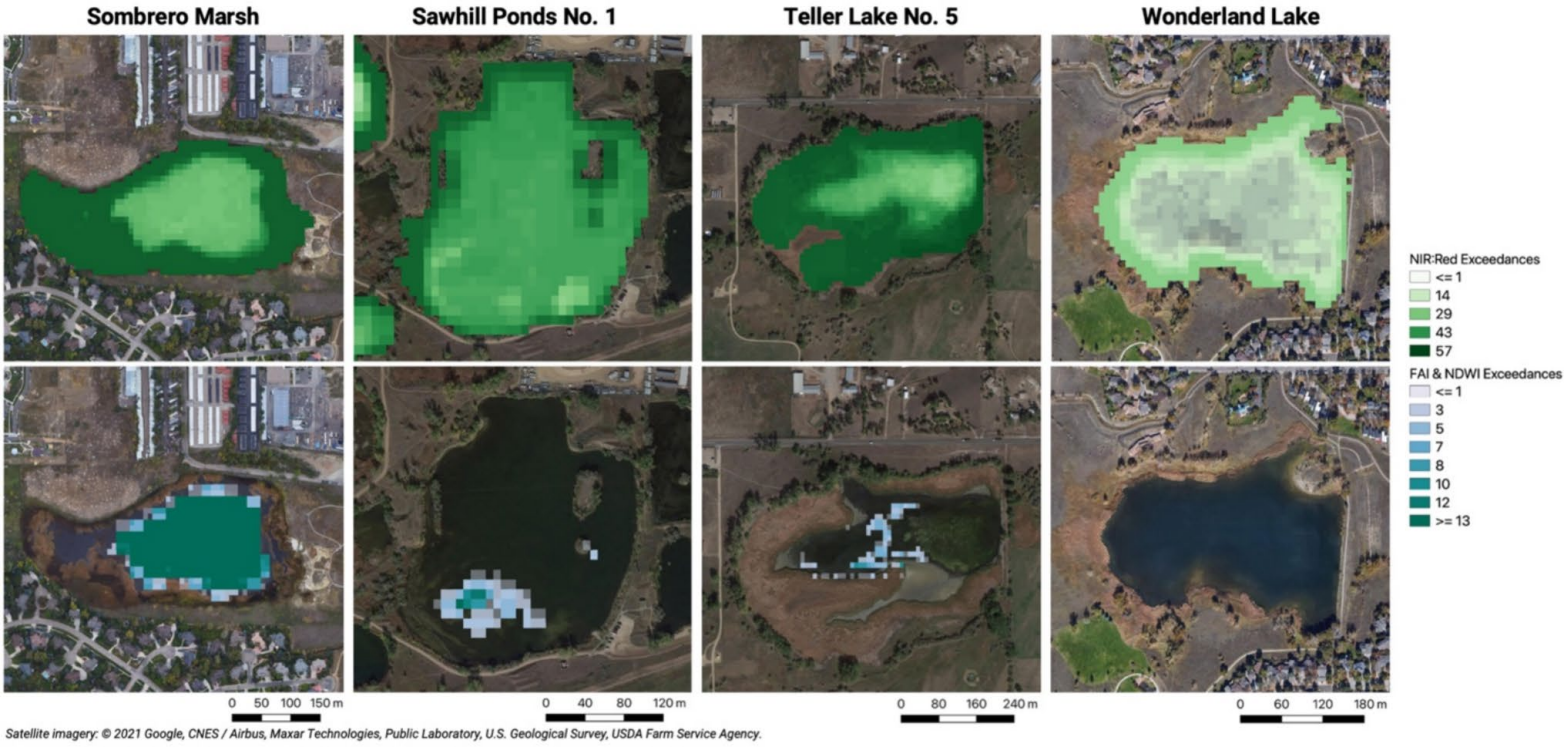
The number of exceedances counted using the NIR:Red threshold (top) and the FAI and NDWI thresholds (bottom) from April 1, 2021, through October 1, 2021, at each waterbody

The exceedance maps provide another outlook on monitoring algal blooms because the greater the number of exceedances, the more persistent a probable algal or cyanobacterial bloom may be (Fig. 3). All four ponds presented consistent evidence of algal growth according to maps showing the number of times each pixel within their boundaries recorded a NIR:Red value greater than 1. In contrast, maps of FAI-NDWI output suggested that three of the four ponds had cyanobacteria present, whereas the time series results above indicated only Sombrero Marsh did. According to the maps for Sombrero Marsh, this pond had marked algal growth and likely cyanobacteria blooms throughout most of its open water area. Sawhill Ponds No. 1 and Teller Lake No. 5 also had likely extensive algal blooms according to their NIR:Red ratio maps, but they expressed a smaller area of potential cyanobacteria compared to Sombrero Marsh. Notably, the area of potential cyanobacteria at these two ponds did not overlap with the edge, near edge, and open water pixels corresponding to the in situ sampling points. Wonderland Lake, in contrast to the other three ponds, had a lower number of NIR:Red values > 1 and no occurrences of potential cyanobacteria according to the FAI-NDWI algorithm.

### In situ limnological results

In situ data collected at each of the ponds are presented in Table 2, with the range noted for each analyte and the date at which the maximum occurred for the analyte (for more information, review Appendix Figs. 10, 11, 12, and 13).

**Table 2:** Summary water quality data. Dates in parentheses indicate the date at which the maximum for the analyte occurred. ND indicates non-detect

Variable	Unit	Sawhill Ponds No. 1	Sombrero Marsh	Teller Lake No. 5	Wonderland Lake
Temperature	°C	20.2–26.7 (2021-06-15)	15.9–25.5 (2021-06-14)	18.8–25.8 (2021-07-26)	17.9–28.0 (2021-06-15)
pH	-	9.5–12.9 (2021-08-10)	8.4–12.0 (2021-08-09)	8.0–10.7 (2021-07-01)	8.0–11.4 (2021-06-30)
Specific conductance	µS/cm	721–798 (2021-08-10)	4734–6986 (2021-06-14)	687–787 (2021-07-26)	1670–1725 (2021-07-13)
Dissolved oxygen	mg/L	3.9–14.0 (2021-06-30)	0.0–20.3 (2021-06-30)	4.8–9.3 (2021-06-14)	6.0–10.0 (2021-06-15)
	%	48.1–165.0 (2021-06-30)	0.4–225.6 (2021-06-30)	55.7–114.2 (2021-06-14)	73.2–124.0 (2021-06-15)
Chlorophyll-a	mg/L	0.3–2.6 (2021-06-30)	9.8–287.3 (2021-08-09)	0.1–18.3 (2021-07-26)	0.3–14.7 (2021-08-10)
Phaeophytin	mg/L	ND	35.0–230.6 (2021-08-09)	ND	0.7–9.0 (2021-08-10)
TIN	µg/L	20.7–82.1 (2021-08-10)	47.7–968.4 (2021-07-26)	12.2–52.5 (2021-07-01)	25.0–71.7 (2021-06-15)
SRP	µg/L	0.7–6.2 (2021-07-27)	5.8–240.0 (2021-06-14)	0.7–5.4 (2021-07-1)	0.4–3.1 (2021-06-30)
Cyanobacteria count	counts/L*	1429–10,000 (2021-07-27)	85,714–3.41E + 7 (2021-07-26)	1429–1.41E + 5 (2021-07-26)	1429–4.64E + 5 (2021-08-10)

*Corrected concentration for the 14% efficiency: $\frac{counts}{L}=\frac{count}{14\%*0.005L}$

Water temperatures fluctuated over time as expected in a shallow water habitat, with warm days bringing about large increases in water temperature. At some locations, the jump in surface water temperature exceeded 5 °C. Across most ponds, the peak surface water temperature occurred in mid-June. pH remained alkaline in all ponds with Sawhill Ponds No. 1 being the most alkaline with a range from 9.47 to 12.87. For Sombrero Marsh, the pH ranged from 8.37 to 11.96, Teller Lake No. 5 pH ranged from 7.99 to 10.65, and Wonderland Lake pH ranged from 7.96 to 11.39. For these ponds, all sampling sites fell within similar pH ranges. Sombrero Marsh had the highest specific conductance values ranging from 4374 to 6986 µS/cm. Wonderland Lake had the second highest values, approximately a quarter of those observed at Sombrero Marsh. Sawhill Ponds No. 1 and Teller Lake No. 5 both had specific conductance readings roughly an order of magnitude lower than the other two ponds. Similarly, Sombrero Marsh had the largest range for DO from 0.03 mg/L (0.4%) to 20.32 mg/L (225.6%). For Teller Lake No. 5, the DO ranged from 4.75 mg/L (55.7%) to 9.33 mg/L (114.2%). Wonderland Lake exhibited a higher range from 5.96 mg/L (73.2%) to 10.0 mg/L (124.0%).

**Fig. 4:**
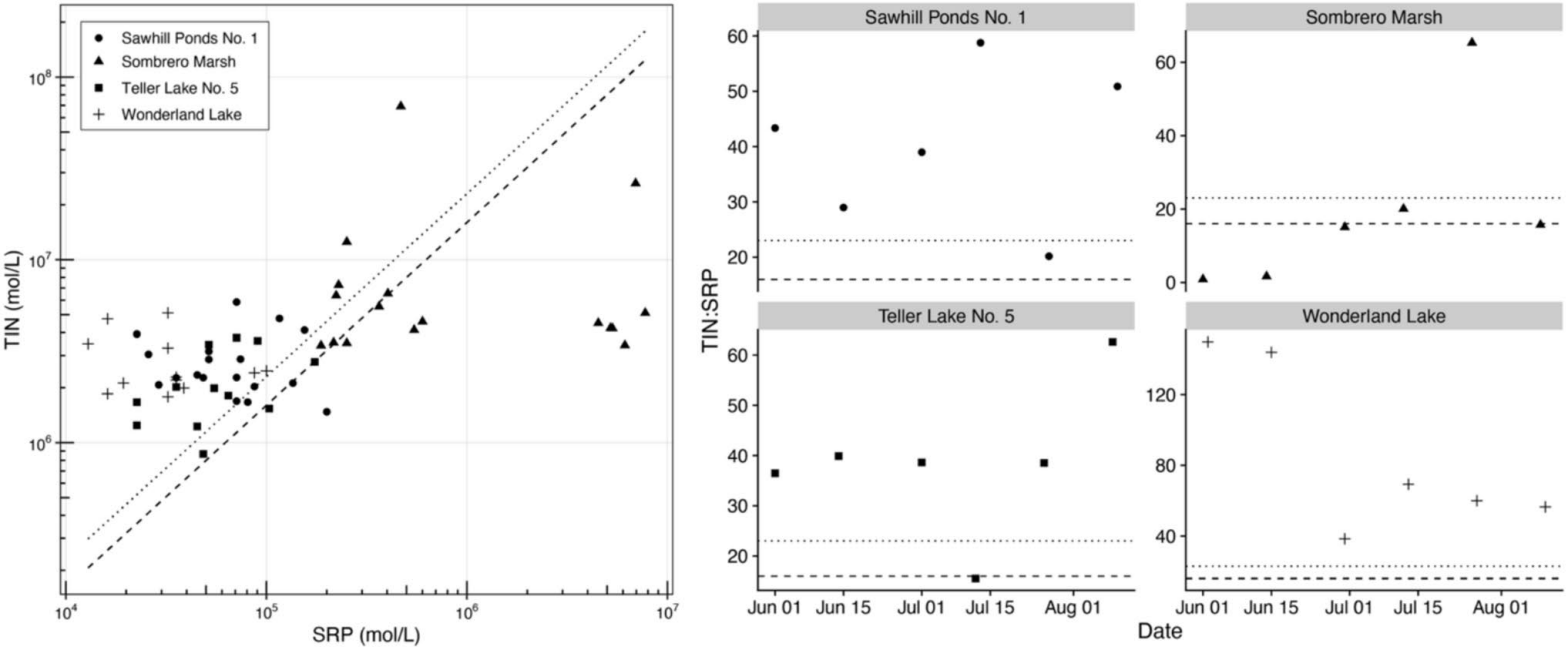
Total inorganic nitrogen (TIN) and soluble reactive phosphorous (SRP) molar concentrations. Dotted line indicates a 23:1 TIN:SRP ratio; dashed line indicates a 16:1 TIN:SRP ratio. Values greater than the ratios are considered P limited environments. Left plot shows all sample locations and sampling dates. Right plot shows an averaged TIN:SRP value for each sampling date over the sampling period

Sombrero Marsh also had the largest ranges for nutrients, as TIN ranged from 48 to 968 µg/L, with its peak in late July primarily fueled by ammonium, and SRP being highest in mid-June. Nitrate ranged from 1.2 to 58 µg/L across all ponds, and nitrite concentrations similarly remained relatively low, with a range of 0 to 1.39 µg/L. The ratio between TIN and SRP did not follow a specific trend over the summer; the ratio varied among waterbodies for similar sampling dates (Fig. 4). Sawhill Ponds No. 1, Teller Lake No. 5, and Wonderland Lake had TIN:SRP ratios greater than 23, which indicates a P-deficiency. Sombrero Marsh only had 2 sampling dates when the TIN:SRP ratio was greater than 23 while most of the summer TIN:SRP ratio was less than 23, indicating a N-deficiency.

**Fig. 5:**
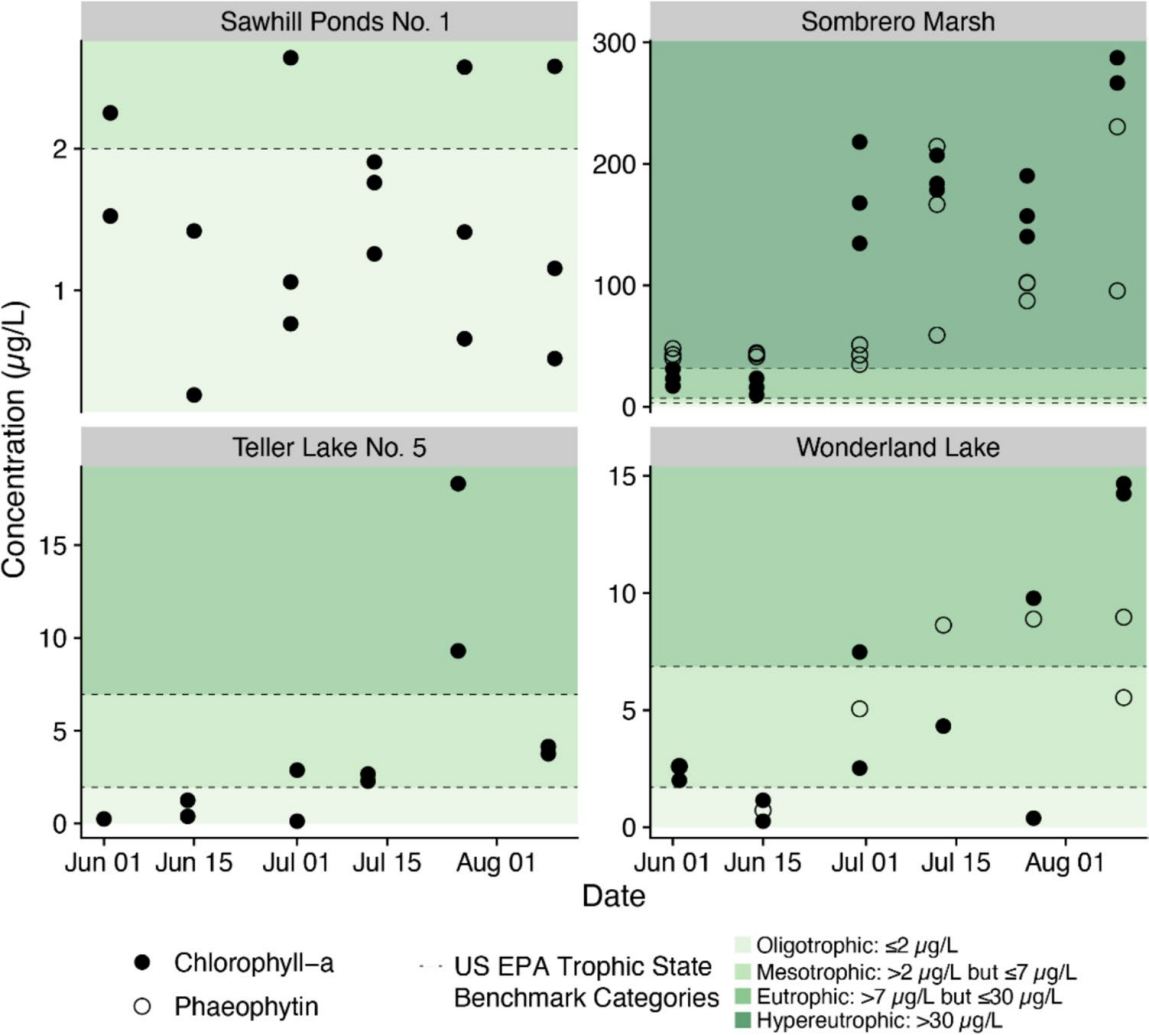
Chlorophyll-a and phaeophytin concentrations at all sampling sites from June to August. The EPA trophic state benchmark ranges are indicated with dotted lines and color blocks. Values below detection limits are omitted. Note: *y*-axis scales vary between panels

Lab analyses indicated that the phaeophytin concentrations were higher in the early summer, with chlorophyll-a dominating later in the summer (Fig. 5). Sombrero Marsh had the highest chlorophyll-a and phaeophytin concentrations among all four ponds, maintaining a hypereutrophic state. Chlorophyll-a values at Sawhill Ponds No. 1 were lower than all the other ponds with maximum concentrations less than 3 µg/L, indicating the least amount of algal growth across all ponds. Teller Lake No. 5 and Wonderland Lake both had maximum chlorophyll-a values less than 20 µg/L, while Sombrero Marsh reached a peak of nearly 300 µg/L in late summer. While Teller Lake No. 5 and Wonderland Lake had relatively lower chlorophyll-a concentrations compared to Sombrero Marsh, these ponds maintained mesotrophic states and even reached eutrophic in the late summer.

**Fig. 6:**
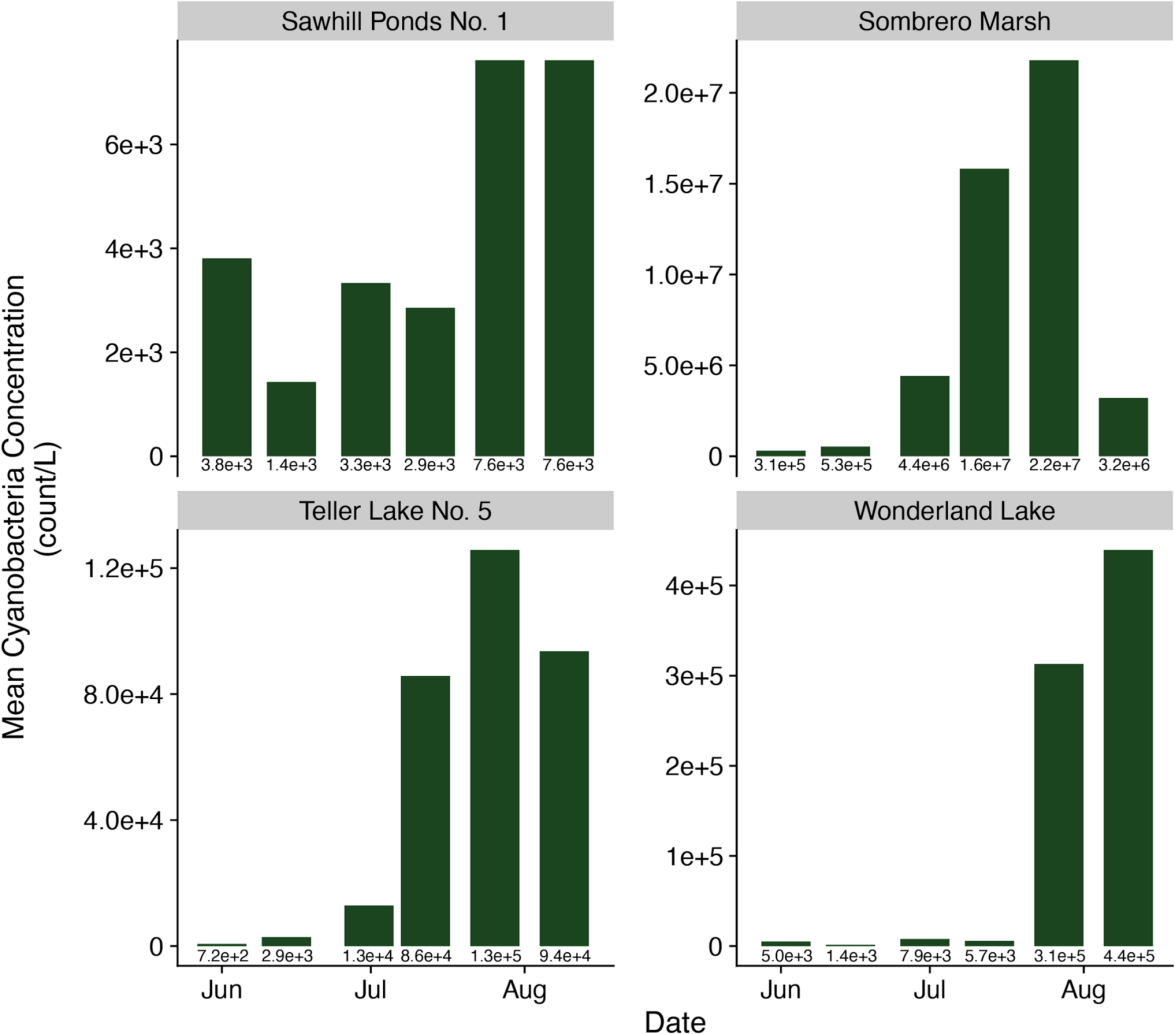
Mean cyanobacteria concentration (count/L) from the FlowCam for each sampling date from June and August. Values are rounded to the nearest whole number and displayed below each bar. Note: *y*-axis scales vary between panels

There were a variety of algal types recorded by the FlowCam, and cyanobacteria were present in all the ponds. Other algal types include green algae, dinoflagellates, and golden algae. Cyanobacteria abundance generally increased throughout the summer either peaking at the end of July or early August, with Sombrero Marsh exhibiting concentrations orders of magnitude higher than the other waterbodies (Fig. 6). Of the identified cyanobacteria, filamentous colonies were the most abundant with *Dolichospermum* (Anabaena) being the dominant genus across the ponds. Other filamentous cyanobacteria genera, e.g., *Oscillatoria* and *Aphanizomenon*, were present in Teller Lake No. 5 and Sombrero Marsh. While less prevalent than filamentous colonies, there were also coccoid cyanobacteria present across the ponds, e.g., *Microcystis.*

### Comparison of remote sensing and in situ results

The water quality in situ data were generally consistent with the remote sensing results, as the trend of increasing NIR:Red values in July through August is reflective of water quality conditions optimal for algal growth and consistent with increasing observed chlorophyll-a concentrations. As indicated by the remote sensing outputs, Sombrero Marsh had the greatest signal of cyanobacteria presence from the FAI-NDWI method and reliable signals of algal biomass from the NIR:Red method throughout the summer months. This high algal abundance is consistent with the water quality data from Sombrero Marsh as it was eutrophic and hypereutrophic throughout the summer with the largest range for specific conductance, dissolved oxygen, and nutrients.

**Fig. 7:**
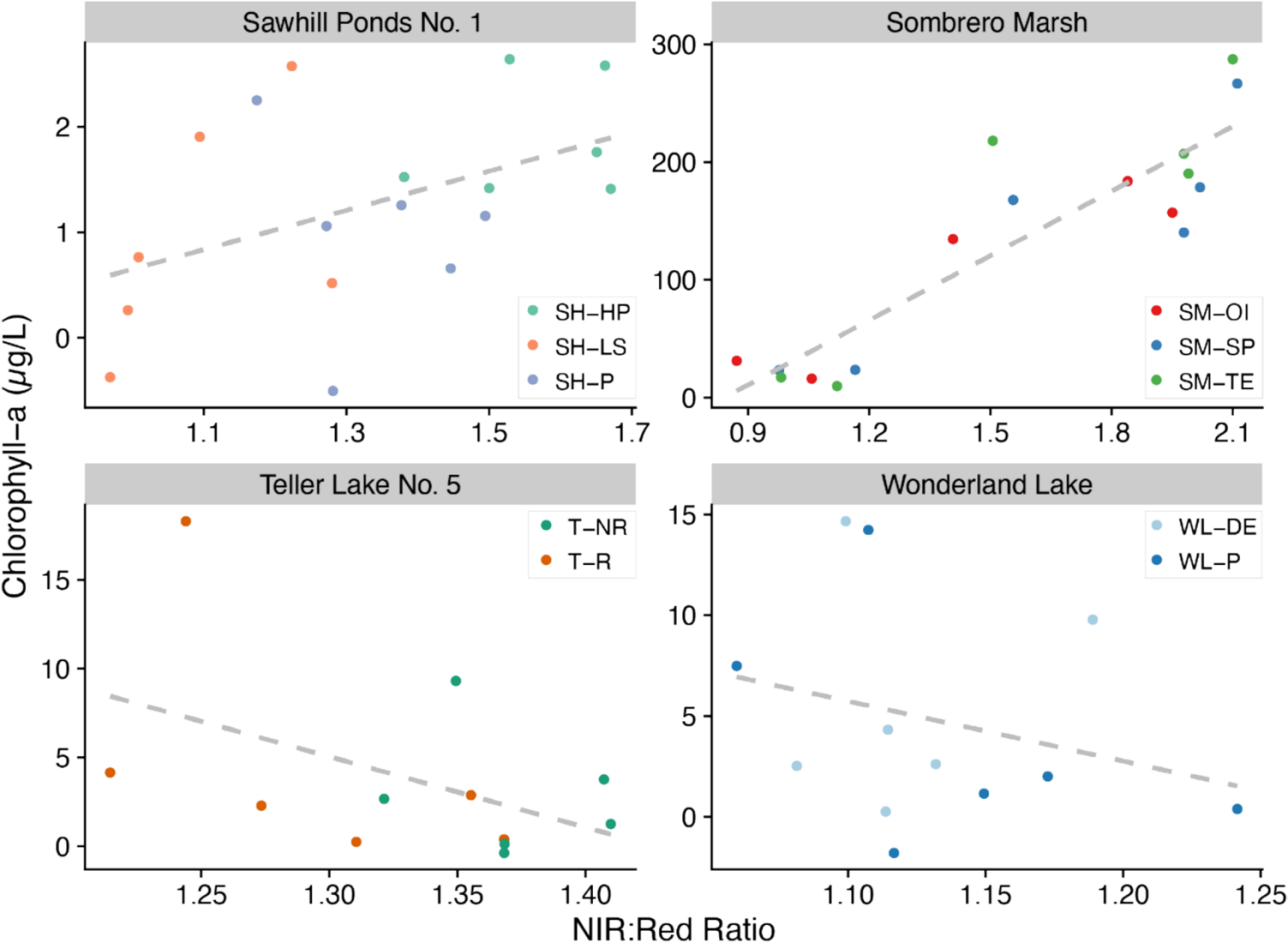
Comparison of the remotely sensed NIR:Red ratio to lab-tested values of chlorophyll-a from the four waterbodies. The grey line is the line of best fit at each waterbody. The slope of the best fit line is only statistically significant (*p* < 0.05) at Sombrero Marsh

We found mixed results when comparing lab-measured chlorophyll-a values to the NIR:Red ratio. Across all ponds, there was a statistically significant positive relationship between NIR:Red and chlorophyll-a. Although there was a large amount of scatter in the values, we computed an *r*^*2*^ of 0.57 and a *p-value* of less than 0.0005. The slope of the best-fit line was 189.2, indicating that an increase in the NIR:Red ratio of 1 was associated with a 189.2 µg/L increase in the chlorophyll-a concentration. However, when we examined the ponds individually (Fig. 7), only chlorophyll-a and NIR:Red values from Sombrero Marsh had a statistically significant relationship (*p* < 0.0005), with an *r*^*2*^ of 0.80 and a slope of 182.9. All other per-waterbody relationships were not statistically significant.

**Fig. 8:**
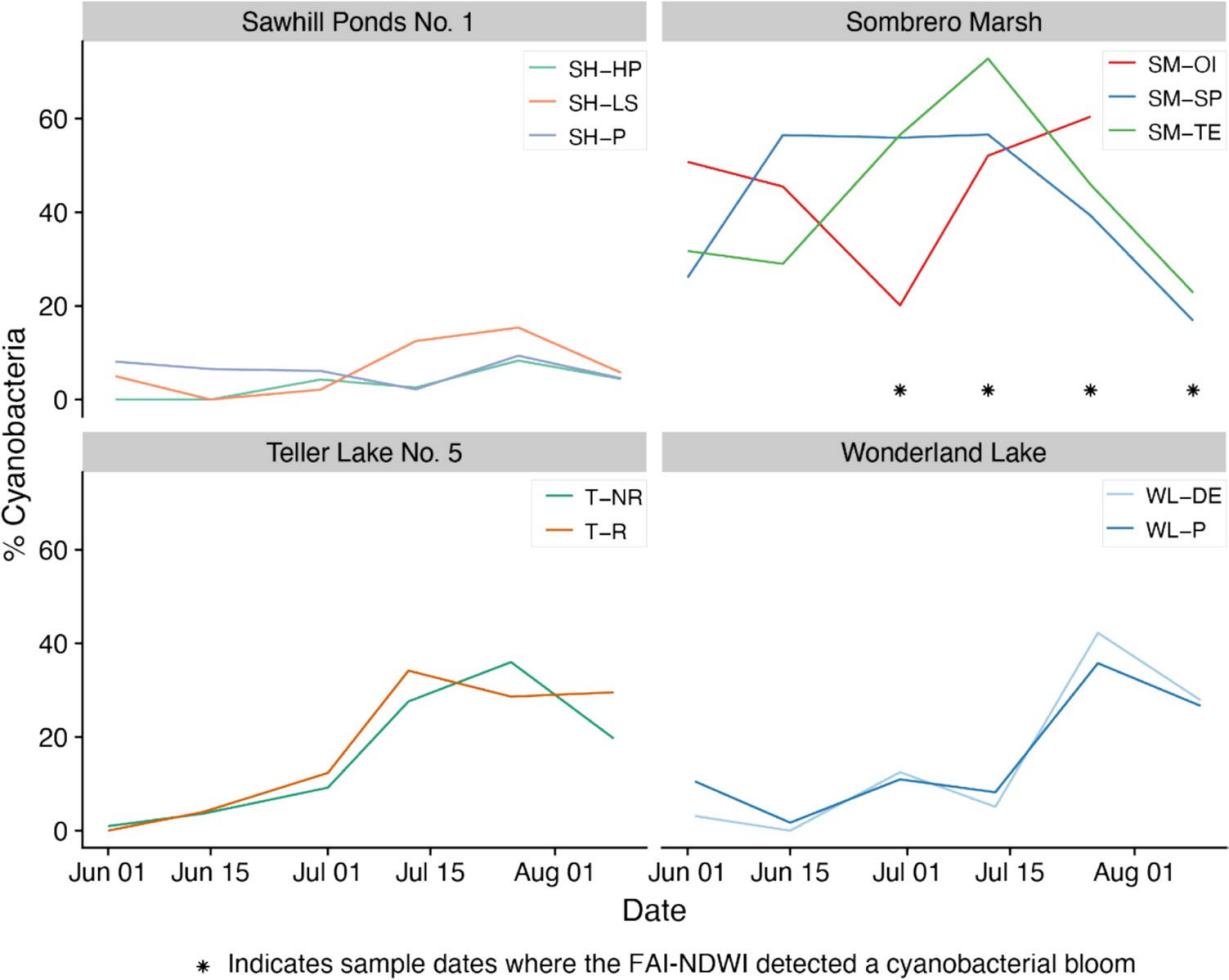
Time series of percent cyanobacteria in water samples. Star points indicate dates where the FAI-NDWI detected a cyanobacterial bloom

Relative to FlowCam-observed cyanobacteria abundance, the remote sensing FAI-NDWI algorithm expressed a 52.5% accuracy. FlowCam results indicate that abundance, calculated as the concentration (count per liter), generally increased throughout the summer either peaking at the end of July or early August (Fig. 8). At the beginning of the sampling season, biomass counts were in the hundreds, and then reached thousands in July for Sombrero Marsh and in late July for Wonderland Lake. Further investigation into cyanobacteria and its presence throughout the sampling period revealed Sombrero Marsh had the highest counts and percentage of cyanobacteria biomass. The FAI-NDWI algorithm correctly predicted 10 true positives and 21 true negatives, which were when the algorithm and FlowCam (cyanobacteria count >  = 5) both indicated cyanobacteria presence or absence, respectively. The algorithm produced 28 false negatives, suggesting no cyanobacteria were present when the FlowCam indicated that they were present. There were no false positives, suggesting the algorithm was relatively conservative and that the cyanobacteria counts from Teller Lake No. 5, Sawhill Ponds No. 1, and Wonderland Lake were difficult to detect.

## Discussion

In this study, the combination of in situ sampling and remote sensing provided an integrated approach to monitoring algal blooms in Boulder OSMP ponds. The addition of water quality sampling enabled a more robust analysis for understanding biogeochemical responses to changing summertime environmental conditions that promote algal growth. Surface water temperatures are responsive to the fluctuations in air temperature at the time of sampling, and these warmer surface water temperatures, alkaline pH values, and higher relative dissolved oxygen (DO) values are indicative of algal bloom development. Alkaline waters could occur due to depletion of dissolved CO_2_ and insufficient reaeration with the atmosphere, a characteristic of eutrophic waterbodies in the summer, allowing the more tolerant cyanobacteria to outcompete other algal species (Zepernick et al. 2021). DO values larger than 100% saturation are indicative of dissolved gases in the surface waters not being in equilibrium with the overlying atmosphere. Two likely causes are (1) active photosynthesis (Burke 1995) and (2) rapid changes in temperature that lead to non-equilibrium with water and air, so the oxygen is “trapped” in the water at time of sampling (Weitkamp & Katz 1980). The TIN:SRP ratio varied among waterbodies for similar sampling dates. Interestingly, Sombrero Marsh only had 2 sampling dates when the ratio was greater than 23, while most of the summer TIN:SRP was less than 23, which can serve as evidence for nitrogen-assimilating cyanobacteria. This imbalance to a N-limited environment happens when there is an abundance of N-fixing taxa (e.g., *Anabaena* and *Aphanizomenon*) (Dolman et al. 2012; Nõges et al. 2008; Paerl et al. 2001).

With the assumption that these ponds have a relatively high residence time which promotes stratification, these conditions provide optimal conditions for algae to grow especially late in the summer season (i.e., July and August). This occurrence was observed with the remote sensing, FlowCam, and water quality results. In general, across all ponds, there was an increase in the concentration of chlorophyll-a with higher NIR:Red values. Of the four ponds, Sombrero Marsh had the greatest in situ chlorophyll-a, over an order of magnitude greater than what was measured in the other ponds. Its remotely sensed NIR:Red ratio values were also generally higher than the other ponds, particularly when evaluating the open water pixels. Furthermore, only for Sombrero Marsh was a significant positive relationship found between the NIR:Red ratio and chlorophyll-a. This indicates that across all sites, the remotely sensed ratio can detect chlorophyll-a concentrations, but only at higher values likely falling within the hypereutrophic range. Therefore, the NIR:Red ratio will likely have considerable uncertainty if used to predict lower chlorophyll-a concentrations in specific small pond-sized waterbodies, even for those that become eutrophic over the summer.

In the laboratory, we observed several types of algae via FlowCam imagery, including cyanobacteria, green, and golden algae as well as dinoflagellates. Similar to the chlorophyll-a analysis, Sombrero Marsh supported the greatest abundance and most frequent occurrences of cyanobacteria when examining FlowCam imagery. Interestingly, the other three ponds all showed evidence of cyanobacteria presence during the 2021 summer sampling season, generally increasing after mid-to-late July. In contrast, however, the FAI-NDWI time-series algorithm tended to be relatively conservative in detecting cyanobacterial presence given that only Sombrero Marsh expressed evidence of cyanobacteria presence in the pixels overlaid with the physical sampling locations. However, the exceedance maps, which cover the waterbodies in their entirety, suggested a different story, with Sawhill Ponds No. 1 and Teller Lake No. 5 both showing potential cyanobacteria blooms as was observed with the FlowCam imagery. For Wonderland Lake, however, neither the remotely sensed time series nor map data indicated cyanobacteria being present. In contrast, FlowCam imagery showed that cyanobacteria were present and additional molecular analyses confirmed that Wonderland Lake had > 10,000 reads of *Microcystis* and *Synechococcus* in the August sampling date (Collins 2023). Nevertheless, this combination of approaches shows a much more complete picture of cyanobacterial patterns than any method used on its own. But this also highlights the shortcomings of sampling and testing a selection of locations—the spatiotemporal variability of algal blooms calls for comprehensive monitoring across space and time.

### Assumptions and limitations

Remote sensing approaches are subject to important assumptions and limitations, such as waterbody size, mixed land-water pixels, and atmospheric effects (cloud cover, haze). We also imposed empirically derived thresholds for determining cyanobacteria blooms. Such thresholds may not work for waterbodies in different geographic regions. For example, we found that the FAI-NDWI maps detected cyanobacterial blooms that the time series analysis did not. This expected behavior, caused by the inherent variability in cyanobacteria patterns, underscores the need for a multi-faceted approach to monitoring HABs. The lack of false positives suggests it is relatively conservative, underreporting potential blooms. Another consideration is that cyanobacteria can move within a water column. Many cyanobacteria species have gas vacuoles that allow them to regulate their buoyancy (Kutser et al. 2008), meaning cyanobacteria can move from the surface to lower depths that satellite sensors may not be able to measure.

The regressions performed on NIR:Red to chlorophyll-a data showed insignificant relationships at all sites except for Sombrero Marsh. This pond had both the highest maximum (287 µg/L) and the largest range in chlorophyll-a concentrations in 2021. These values put Sombrero Marsh on the low side of data presented in previous literature. Tebbs et al. (2013), for example, regressed chlorophyll-a concentrations approaching 700 µg/L against band ratio values nearing 3.5 as computed from remote sensing data. Similarly, Oyama et al. (2015) evaluated remote sensing output from waterbodies with chlorophyll-a concentrations between 174 and 21,736 µg/L. This is important because water absorbs much of the incoming shortwave radiation emitted by the sun, which causes satellite sensors to report a greater amount of noise relative to the signal returned by low over-water reflectances. Functionally, this means waterbodies with denser algal blooms (i.e., greater chlorophyll-a concentrations), such as Sombrero Marsh, return a more reliable signal. The NIR:Red ratio is therefore a less reliable indicator of absolute chlorophyll-a values in waterbodies with low chlorophyll-a concentrations.

There are existing satellite instruments and algorithms to track larger, denser, more persistent blooms in marine environments and large freshwater systems (e.g., MODIS (NASA) and Sentinel-3 (ESA)). The recently launched Plankton, Aerosol, Cloud, ocean Ecosystem (PACE) satellite (NASA) also focuses on marine environments with additional dedicated technology to provide phytoplankton concentration. However, across these satellite technologies, applicability and spatial resolution is a persistent problem for HABs monitoring in pond-sized waterbodies because the technology itself is not suitable for waterbodies that are often smaller than a single pixel output (e.g., the PACE satellite has a 1.2-km^2^ spatial resolution; the largest waterbody in this study was 6.1 ha, which is about 0.061 km^2^).

Determining the toxicity of a sample remains challenging, as neither microscopy nor remote sensing can confirm whether the cyanobacteria are toxic. Only lab-based toxicity analyses can determine this characteristic. This is important given that cyanotoxins can remain in the water even after a bloom has visually subsided. Since minimizing human exposure to these toxins is a public health priority, alternative approaches can be used to assess algal community composition as a proxy for potential risk of cyanotoxins. For instance, using FlowCam imagery allows for rapid identification of algal types (Collins 2023), but requires access to the instrumentation. Environmental DNA (eDNA) analysis provides a deeper and more comprehensive review of algal species present, but it is more resource intensive. While these methods cannot directly confirm toxicity, they remain valuable tools for identifying potentially harmful cyanobacteria before more targeted lab-based toxicity analyses are conducted.

### Looking ahead

From a public health context, the false negatives produced from these remote sensing methods for these ponds warrant attention. The FAI-NDWI algorithm did not reliably resolve cyanobacteria. In practice, this could mean water managers may not know that there are cyanobacteria present in their pond-sized waterbodies, which can have public health consequences. The NIR:Red algorithm, on the other hand, was able to reliably detect algal biomass on the water surface and the exceedance maps showed areas more prone to algal accumulation and extent but could not resolve bloom intensity. This calls for improved algorithms. Having said that, many of the efforts in refining algorithms are regionally specific, making it difficult to scale, and HABs monitoring research suffers from geographic imbalances and a shared, approachable methodology (Feng et al. 2024). Perhaps instead of developing regionally specific algorithms, we should look towards sharing and integrating in situ and remote sensing data from smaller inland waterbodies, like ponds. This strategy could help build a more comprehensive database to overcome these limitations in hopes of developing algorithms that can overcome the spatiotemporal challenge of monitoring HABs in these smaller waterbodies.

On the management level, it could be enough to use the NIR:Red algorithm to just know when and where potential algal blooms will be more persistent, which offers information on when to conduct in situ sampling and possibly consider closing access to the waterbody or discontinue use as a water supply. We proposed a mixed approach that can be applied in a variety of ways. For example, one could apply the NIR:Red ratio to inform collection of grab samples for microscopy to identify if cyanobacteria are present. However, in situ monitoring and microscopy during the summertime may be advisable for pond-sized waterbodies of importance and for which remote sensing has been found to be unreliable.

## Conclusion

This study tracked the evolution of algal blooms in four Boulder OSMP ponds using in situ testing, laboratory analyses, and remote sensing data primarily to answer the question if remote sensing can be used as a reliable monitoring tool for algal blooms in pond-sized waterbodies. The remote sensing data showed an increasing NIR:Red ratio from spring to late summer, corresponding to algal growth at the four ponds. Lab-measured chlorophyll-a concentrations increased from spring through late summer, where the peak concentrations were an order of magnitude higher at Sombrero Marsh than at the other waterbodies. Sombrero Marsh generally maintained a hypereutrophic state throughout the summer. Because of this, the NIR:Red ratio had a positive, statistically significant relationship with chlorophyll-a concentrations only at Sombrero Marsh. The FAI-NDWI algorithm underpredicted cyanobacterial blooms and recorded no false positives but one false negative of concern. The time series values of the FAI-NDWI algorithm indicated cyanobacteria presence only at sampling locations in Sombrero Marsh, and maps from the algorithm output showed additional cyanobacterial blooms at Teller Lakes No. 5 and Sawhill Ponds No. 1. Nonetheless, the more definitive FlowCam imagery showed cyanobacteria at all the ponds with it most concentrated at Sombrero Marsh.

The findings of this work highlight the utility of remote sensing for identifying algal blooms in smaller waterbodies with important caveats. Monitoring algal blooms will likely require a multi-step approach that leverages the best aspects of each explored method. Remote sensing is a cost-effective tool that can quickly provide information on the spatiotemporal patterns of algal blooms and HABs, while in situ sampling and subsequent analyses can produce more granular and definitive information on water quality and algae type. Both methods provide complementary insights for algal growth patterns, but using just remote sensing will not capture the entire scope and may not be sufficient for protecting public health unless improvements are made that focus on the unique attributes of pond-sized waterbodies.

## Figures and Tables

**Fig. 9:**
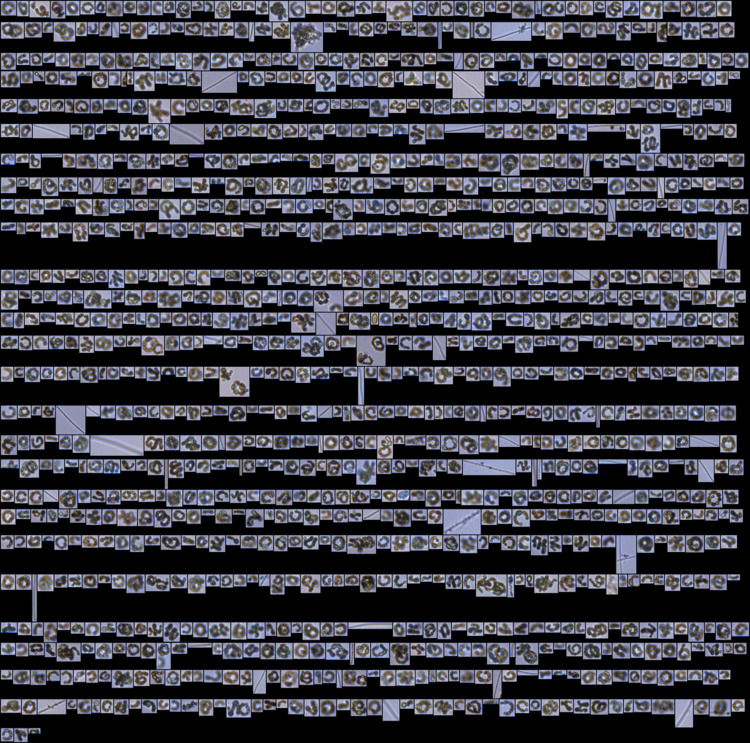
FlowCam output (× 10 magnification) displaying different types of cyanobacteria, diatoms, other algae types, and possibly pollen. Example from 2021-07-12 at Stinky Peat, Sombrero Marsh (one of 16 photo outputs from the 5.0 mL water sample)

**Fig. 10:**
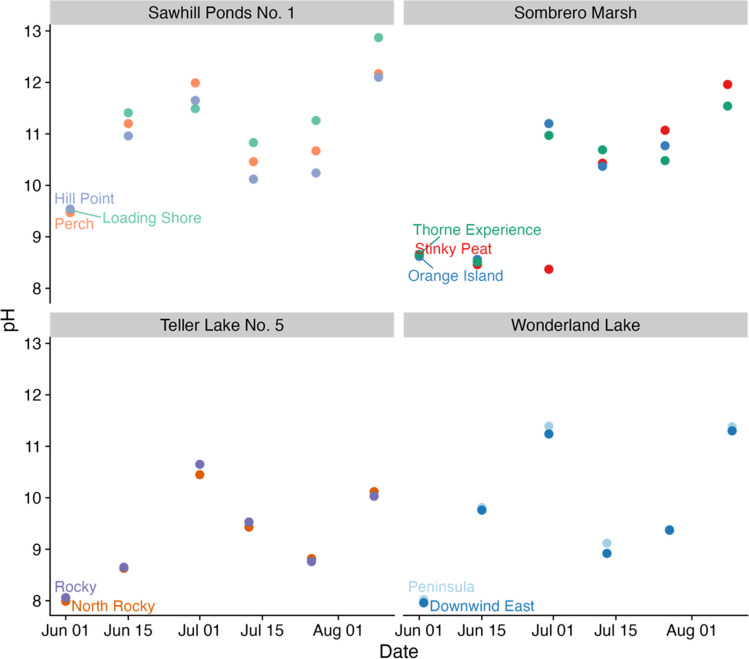
pH measured in situ at each waterbody and sampling location

**Fig. 11:**
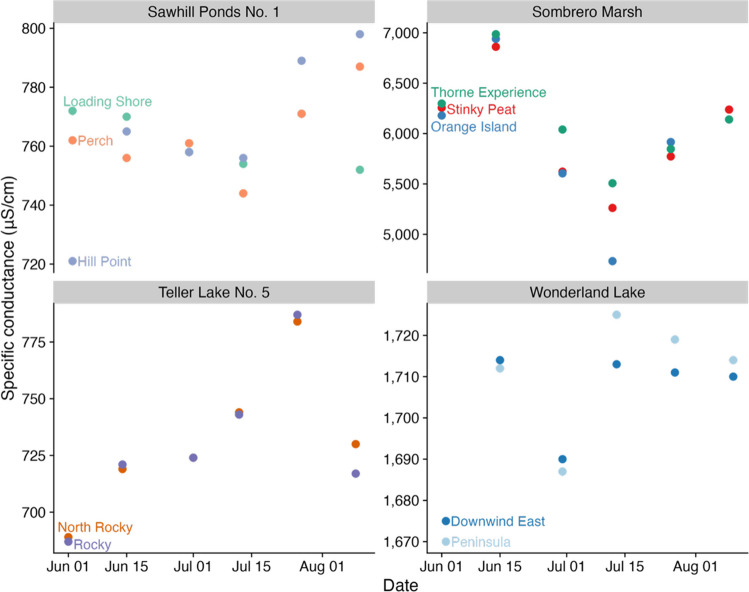
Specific conductance measured in situ at each waterbody and sampling location

**Fig. 12:**
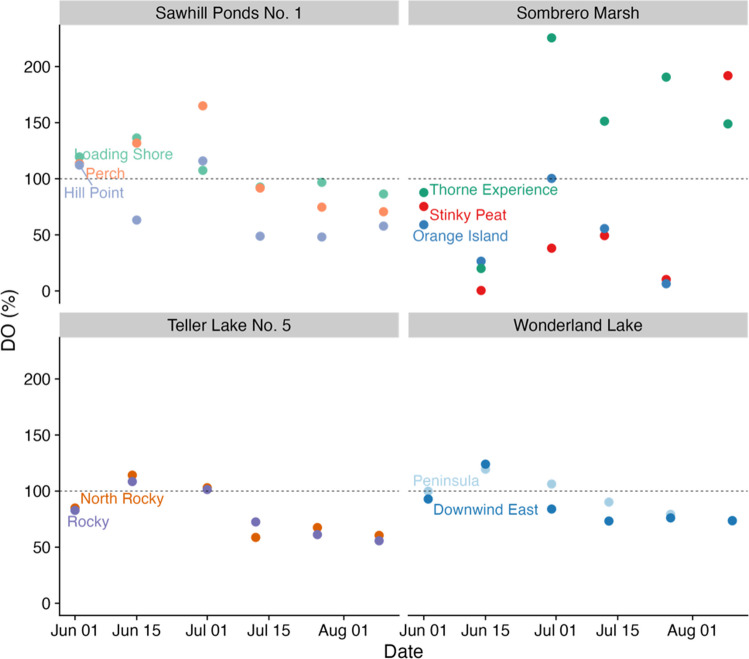
Dissolved oxygen (DO) in percentage measured in situ at each waterbody and sampling location

**Fig. 13:**
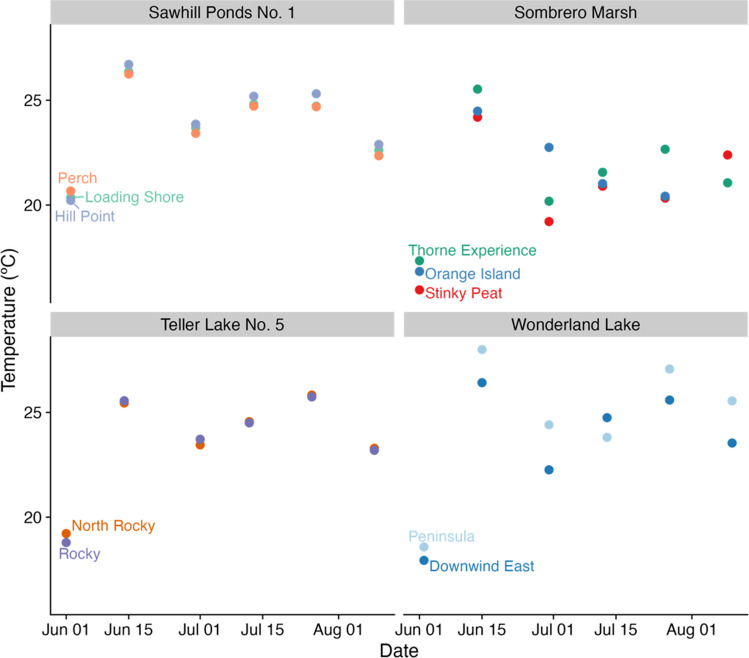
Water temperature measured in situ at each waterbody and sampling location

## Data Availability

The datasets generated and/or analyzed during the current study are available from the corresponding author on reasonable request.

## Appendix

### Locations and sampling dates

**Table 3:** Locations and sampling dates for each sampling site. (Note: missing data from 2021–08-09 for Orange Island in Sombrero Marsh—we were unable to access the sampling location due to overgrown conditions)

Waterbody	Sampling site	Coordinates	Sampling dates
Sawhill Pond No. 1	Loading Shore	40°02′22.8″N 105°11′07.6″W	2021-06-01, 2021-06-15, 2021-07-01, 2021-07-13, 2021-07-27, 2021-08-10
	Perch	40º02′21.68″ N 105º11′11.77″ W	2021-06-01, 2021-06-15, 2021-07-01, 2021-07-13, 2021-07-27, 2021-08-10
	Hill Point	40º02′25.72″ N 105º11′14.88″ W	2021-06-01, 2021-06-15, 2021-07-01, 2021-07-13, 2021-07-27, 2021-08-10
Sombrero Marsh	Stinky Peat	40º00′43.15″ N 105º12′17.87″ W	2021-06-01, 2021-06-14, 2021-06-30, 2021-07-12, 2021-07-26, 2021-08-09
	Orange Island	40º00′40.13″ N 105º12′17.54″ W	2021-06-01, 2021-06-14, 2021-06-30, 2021-07-12, 2021-07-26
	Thorne Experience	40º00′44.40″ N 105º12′22.22″ W	2021-06-01, 2021-06-14, 2021-06-30, 2021-07-12, 2021-07-26, 2021-08-09
Teller Lake No. 5	North Rocky	40º02′20.68″ N 105º08′09.77″ W	2021-06-01, 2021-06-14, 2021-07-01, 2021-07-12, 2021-07-26, 2021-08-09
	Rocky	40º02′17.68″ N 105º08′09.74″ W	2021-06-01, 2021-06-14, 2021-07-01, 2021-07-12, 2021-07-26, 2021-08-09
Wonderland Lake	Peninsula	40º03′2″ N 105º17′14″ W	2021-05-20, 2021-06-02, 2021-06-15, 2021-06-30, 2021-07-13, 2021-07-27, 2021-08-10
	Downwind East	40º02′60″ N 105º17′12″ W	2021-05-20, 2021-06-02, 2021-06-15, 2021-06-30, 2021-07-13, 2021-07-27, 2021-08-10

### FlowCam

Satellite specifications

**Table 4:** Sentinel-2 band information. Note: there are slight differences, typically less than 10 nm, between Sentinel- 2 A and 2B (https://sentinel.esa.int/web/sentinel/user-guides/sentinel-2-msi/resolutions/radiometric)

Band	Name	Spectral range (nm)	Spatial resolution (m)
1	Coastal Aerosol	432–453	60
2	Blue	458–523	10
3	Green	543–578	10
4	Red	650–680	10
5	Red Edge 1	698–713	20
6	Red Edge 2	733–748	20
7	Red Edge 3	773–793	20
8	NIR	785–899	10
8a	Narrow NIR	855–875	20
9	Water Vapor	935–955	60
10	SWIR1	1358–1389	60
11	SWIR2	1565–1655	20
12	SWIR3	2100–2280	20

Water quality data
